# Probabilistic White Matter Atlases of Human Auditory, Basal Ganglia, Language, Precuneus, Sensorimotor, Visual and Visuospatial Networks

**DOI:** 10.3389/fnhum.2017.00306

**Published:** 2017-06-19

**Authors:** Teresa D. Figley, Behnoush Mortazavi Moghadam, Navdeep Bhullar, Jennifer Kornelsen, Susan M. Courtney, Chase R. Figley

**Affiliations:** ^1^Department of Radiology, University of Manitoba Winnipeg, MB, Canada; ^2^Division of Diagnostic Imaging, Health Sciences Centre Winnipeg, MB, Canada; ^3^Neuroscience Research Program, Kleysen Institute for Advanced Medicine Winnipeg, MB, Canada; ^4^Department of Physiology and Pathophysiology, University of Manitoba Winnipeg, MB, Canada; ^5^St. Boniface Hospital Research, Catholic Health Corporation of Manitoba Winnipeg, MB, Canada; ^6^Department of Psychological and Brain Sciences, Johns Hopkins University Baltimore, MD, United States; ^7^Solomon H. Snyder Department of Neuroscience, Johns Hopkins University Baltimore, MD, United States; ^8^F.M. Kirby Research Center for Functional Brain Imaging, Kennedy Krieger Institute Baltimore, MD, United States; ^9^Biomedical Engineering Graduate Program, University of Manitoba Winnipeg, MB, Canada

**Keywords:** atlas, brain, connectivity, connectome, diffusion, MRI, white matter

## Abstract

**Background**: Despite the popularity of functional connectivity analyses and the well-known topology of several intrinsic cortical networks, relatively little is known about the white matter regions (i.e., structural connectivity) underlying these networks. In the current study, we have therefore performed fMRI-guided diffusion tensor imaging (DTI) tractography to create probabilistic white matter atlases for eight previously identified functional brain networks, including the Auditory, Basal Ganglia, Language, Precuneus, Sensorimotor, Primary Visual, Higher Visual and Visuospatial Networks.

**Methods**: Whole-brain diffusion imaging data were acquired from a cohort of 32 healthy volunteers, and were warped to the ICBM template using a two-stage, high-dimensional, non-linear spatial normalization procedure. Deterministic tractography, with fractional anisotropy (FA) ≥0.15 and deviation angle <50°, was then performed using the Fiber Association by Continuous Tracking (FACT) algorithm, and a multi-ROI approach to identify tracts of interest. Regions-of-interest (ROIs) for each of the eight networks were taken from a pre-existing atlas of functionally defined regions to explore all ROI-to-ROI connections within each network, and all resulting streamlines were saved as binary masks to create probabilistic atlases (across participants) for tracts between each ROI-to-ROI pair.

**Results**: The resulting functionally-defined white matter atlases (i.e., for each tract and each network as a whole) were saved as NIFTI images in stereotaxic ICBM coordinates, and have been added to the UManitoba-JHU Functionally-Defined Human White Matter Atlas (http://www.nitrc.org/projects/uofm_jhu_atlas/).

**Conclusion**: To the best of our knowledge, this work represents the first attempt to comprehensively identify and map white matter connectomes for the Auditory, Basal Ganglia, Language, Precuneus, Sensorimotor, Primary Visual, Higher Visual and Visuospatial Networks. Therefore, the resulting probabilistic atlases represent a unique tool for future neuroimaging studies wishing to ascribe voxel-wise or ROI-based changes (i.e., in DTI or other quantitative white matter imaging signals) to these functional brain networks.

## Introduction

Cerebral white matter is comprised of myelinated axons that transmit signals between different brain regions, and the importance of these connections is underscored by the severe and wide-spread deficits that arise when they are compromised (e.g., due to traumatic injury, stroke or disorders such as Multiple Sclerosis; Filley, 1998; Schmahmann et al., 2008). However, unlike the gray matter, which has been well mapped, relatively little is known about white matter topology or how particular white matter regions (or sets of regions) correspond to specific brain functions. One approach to tackling this problem is to parcellate and map the white matter using various methods.

Historically, white matter region-of-interest (ROI) analyses have relied on anatomical brain segmentations that are either drawn manually (on an *ad hoc* basis), or imported from an existing brain atlas. As a result, considerable effort has been placed on developing detailed anatomical white matter atlases, such as the well-known JHU “Adam” and “Eve” atlases (Oishi et al., 2008, 2009). Nevertheless, there are several inherent limitations to defining white matter ROIs anatomically. Even in highly-parcellated white matter atlases (such as the aforementioned JHU Eve Atlas), many of the ROIs are relatively large. Therefore, although subsequent analyses may be sensitive to diffuse or global characteristics of the underlying white matter within a given tract, they will not likely be sensitive to small, localized changes (e.g., focal lesions due to encephalitis, radiation necrosis, or Multiple Sclerosis; Djamanakova et al., 2014). Moreover, investigators seeking to examine the structural correlates of individual differences (e.g., within the cognitive, affective, or psychomotor domains) or functional deficits (e.g., within or between populations with particular symptoms) are faced with the dilemma of having to choose *a priori* which white matter region (or set of regions) might be related to the function/symptom/domain of interest. Finally, and as a corollary of the aforementioned limitations, there is a high likelihood that some of the larger anatomically-defined ROIs will span white matter regions underlying multiple neural functions—meaning that even if white matter differences are observed, they may not correspond to differences in specific behaviors, symptoms, or deficits.

One way to address these limitations is to leverage our knowledge about how the brain is organized into functionally-connected networks that are known to be associated with specific neural functions (e.g., sensory, motor, cognitive, etc.; Bressler and Menon, 2010; van den Heuvel and Hulshoff Pol, 2010; Rosazza and Minati, 2011; Smith et al., 2013). Using this approach, our group has recently released a set of functionally-defined white matter atlases for the dorsal and ventral Default Mode, left and right Executive Control, and anterior and posterior Salience Networks (Figley et al., 2015)^1^. These atlases were created using similar methods to those implemented in the creation of the JHU Eve atlas (Oishi et al., 2009), but rather than performing tractography between anatomically-defined gray matter structures, tractography was instead performed between functionally-defined nodes within well-known brain networks (Shirer et al., 2012)^2^. Since these nodes have been defined and grouped using resting state functional connectivity—as opposed to anatomically-defined features or landmarks such as sulci or gyri—an important difference compared to most previous atlases is that the resulting white matter tracts represent structural connections within functional brain networks, rather than traditional white matter connections such as the “superior longitudinal fasciculus”, etc. that have been anatomically constrained. While there is not necessarily a one-to-one correspondence between functional connectivity and anatomical connections, delineating white matter “tracts” based on functional connectivity may enable a better understanding of structure-function relationships.

Using this approach, the goal of the work reported in the current manuscript was to expand our existing set of functionally-defined white matter atlases to include several additional resting state brain networks, including the: (1) Auditory Network (AN); (2) Basal Ganglia Network (BGN); (3) Language Network (LN); (4) Precuneus Network (PN); (5) Sensorimotor Network (SMN); (6) Primary Visual Network (PVN); (7) Higher Visual Network (HVN); and (8) Visuospatial Network (VSN) (Shirer et al., 2012). As a result, future research will be able to examine the structural and functional integrity of the cortical regions within each of these networks, as well as the structural integrity of the white matter pathways that connect them. In particular, it will allow direct comparisons between structural and functional connectivity within these networks, and facilitate both group-wise (e.g., patients vs. healthy controls) and/or regression-based analyses (e.g., with behavioral performance or any other independent variable) in a much more hypothesis-driven manner, based on the known functions of each identified network. Therefore, we hope that these atlases will provide further insights into normal brain-behavior relationships, as well as the functional consequences of brain aging, injury and disease.

## Materials and Methods

### Study Participants

The current analyses were conducted using the same dataset reported in our previous article (Figley et al., 2015). Briefly, the sample included 32 neurologically healthy volunteers (16 males and 16 females; age = 29.9 ± 10.7 years), with no self-reported history of neurological injury/disease, psychiatric illness, or substance abuse. The Johns Hopkins University Institutional Review Board approved the study; and all participants, who were financially compensated for their participation, provided written informed consent prior to enrollment.

### Data Acquisition and Analysis

All data acquisition and analysis methods have been thoroughly described in our previous article (Figley et al., 2015), and thus are only briefly outlined here. All MRI data were acquired on a 3T Philips Achieva system equipped with a 32-channel SENSE head coil (Philips Healthcare, Best, Netherlands). T_1_-weighted anatomical images were obtained using a 3D MP-RAGE pulse sequence (TR = 7.93 ms; TE = 3.66 ms; Flip Angle = 8°; SENSE Factor = 2.4; FOV = 212 mm × 150 mm × 172 mm; Spatial Resolution = 1.00 mm × 1.00 mm × 1.00 mm). Both T_2_-weighted (TR = 4162 ms; TE = 80 ms; Flip Angle = 90°; SENSE Factor = 2; FOV = 212 mm × 154 mm × 212 mm; Spatial Resolution = 1.10 mm × 1.10 mm × 2.20 mm) and T_2_-weighted Fluid Attenuated Inversion Recovery (TR = 11,000 ms; TI = 2800 ms; TE = 120 ms; Refocusing Angle = 120°; SENSE Factor = 1.75; FOV = 230 mm × 149 mm × 184 mm; Spatial Resolution = 1.00 mm × 1.20 mm × 5.00 mm) images were also acquired and assessed by a board-certified radiologist to confirm that none of the participants had structural brain abnormalities or pathologies. Finally, a spin-echo echo-planar imaging sequence was used to acquire diffusion-weighted data (number of diffusion-encoding gradients = 30; b-value = 700 s/mm^2^; number of reference images without diffusion-weighting = 5 (b-value = 0 s/mm^2^); TR = 6904 ms; TE = 69 ms; Flip Angle = 90°; SENSE Factor = 2.5; FOV = 212 mm × 212 mm; Acquired Matrix Dimensions = 96 × 96; Reconstructed Matrix Dimensions = 256 × 256; Number of Transverse Slices = 70 (no gap); Slice Thickness = 2.2 mm).

**Figure 1:**
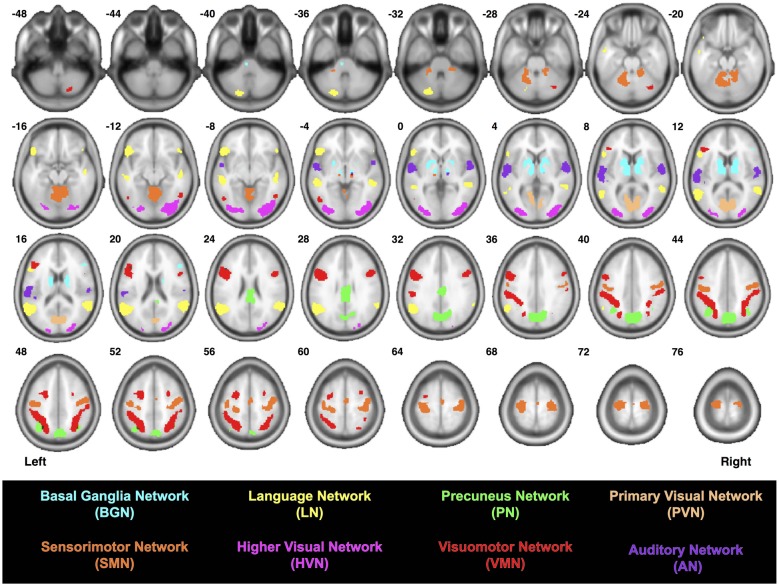
Locations of the functionally-defined nodes within each previously-reported brain network (Shirer et al., 2012).

Images for each participant were processed using a multi-stage analysis pipeline (see Figure 1 from Figley et al., 2015) to: (1) coregister the diffusion-weighted and mean *b* = 0 s/mm^2^ images; (2) correct for motion and eddy current distortions; (3) reorient the gradient direction for each diffusion-weighted image; (4) generate the six tensor images (Landman et al., 2007); (5) skull-strip the coregistered mean *b* = 0 s/mm^2^ image (and apply the mask to the six tensor images); (6) resample all of the skull-stripped images to 1.0 mm^3^ resolution; (7) normalize the data to the “JHU_MNI_SS_b0_ss” template (Mori et al., 2008) in Montreal Neurological Institute (MNI) space (Mazziotta et al., 1995) using high-dimensional, nonlinear warping (Beg et al., 2005) with cascading degrees of nonlinearity (Ceritoglu et al., 2009); and (8) perform whole-brain deterministic tractography in DTIStudio (Jiang et al., 2006) using the Fiber Association by Continuous Tracking (FACT) algorithm (FA > 0.15 and Angle < 50°) and an exhaustive search approach (Mori et al., 1999; Xue et al., 1999). Using a previously reported functional connectivity atlas (Shirer et al., 2012)^3^, a multi-ROI approach was used to constrain the whole-brain tractography data by isolating the streamlines between each pair of functionally-defined nodes (i.e., all possible connections) within the AN, BGN, LN, PN, SMN, PVN, HVN and VSN (Figure 1 and Supplementary Videos 1–8).

Since the AN consists of three nodes (3 ROI-to-ROI combinations), the BGN consists of five nodes (10 ROI-to-ROI combinations), the LN consists of seven nodes (21 ROI-to-ROI combinations), the PN consists of four nodes (6 ROI-to-ROI combinations), the SMN consists of six nodes (15 ROI-to-ROI combinations), the PVN consists of two nodes (1 ROI-to-ROI combination), the HVN consists of two nodes (1 ROI-to-ROI combination) and the VSN consists of 11 nodes (55 ROI-to-ROI combinations), a total of 112 ROI-to-ROI combinations were assessed for each of the 32 participants—yielding a total of 3584 tractography analyses. For each of these, data were visually inspected to identify participants for whom streamlines were present, and all resulting streamlines were saved as binary maps in MNI space. Group probability maps (aka, “probabilistic connectomes”) were then generated for each of the 112 functionally-defined tracts for which streamlines were identified in at least 8/32 participants. This was achieved by adding together the binary maps for each participant (i.e., for a given ROI-to-ROI connection) and dividing by the number of participants. Image intensities for each of the probabilistic connectomes therefore range between 0 and 1 (i.e., where no participants or all 32 participants had streamlines, respectively).

The volume of white matter associated with each resting state network was then calculated by adding all of the group probability maps together for each tract and then multiplying the number of voxels with intensity >0 by the voxel size (i.e., 1 mm^3^). After creating binary masks of each overall connectome (i.e., a combination of all the functionally-defined tracts within each network), we then calculated the amount of spatial overlap between the white matter regions assigned to each network and report these in terms of both actual volumes and normalized ratios (relative to the size of each network), as previously reported (Figley et al., 2015). These results therefore indicate the amount of spatial overlap between a given network and each of the other networks.

Finally, 3D renderings of both the nodes within each network and the resulting white matter group probability maps were overlaid on the JHU_MNI_SS template (Mori et al., 2008) using the Volume and Volume Rendering tools within 3D Slicer (Brigham and Women’s Hospital, Boston, MA, USA; Fedorov et al., 2012)^4^, as previously reported (Figley et al., 2015).

## Results

**Figure 2:**
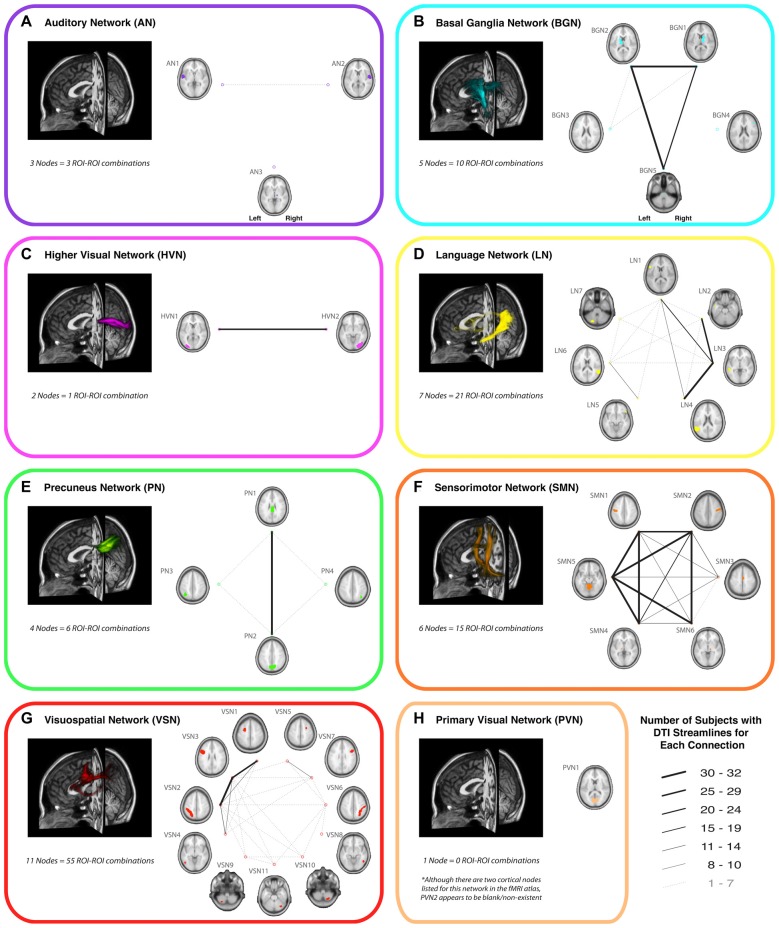
A representative view of the white matter tracts (left) and the connection counts (right) for each functionally-defined white matter tract in **(A)** the Auditory Network (AN), **(B)** the Basal Ganglia Network (BGN), **(C)** the Higher Visual Network (HVN), **(D)** the Language Network (LN), **(E)** the Precuneus Network (PN), **(F)** the Sensorimotor Network (SMN), **(G)** the Visuospatial Network (VSN) and **(H)** the Primary Visual Network (PVN). The nodes within each (Shirer et al., 2012) are shown on axial brain slices (at their center-of-mass), and the connection counts for each tract (i.e., the numbers of participants with tractography streamlines identified between each ROI-to-ROI pair) are represented by the weight of lines between the respective nodes. Note that all axial slices are displayed in neurological convention.

Of all the 112 functionally-defined white matter connections assessed in the current study (via deterministic tractography), some repeatedly yielded DTI streamlines across participants, while others did not. In order to quantify this, the connection counts—i.e., the number of participants exhibiting at least one streamline—for each connection (within each network) are depicted in Figure 2. Interestingly, of the networks containing corresponding bilateral regions, some showed a high degree of symmetry in terms of the tracts with the highest connection counts (i.e., BGN and SMN), while others exhibited a distinct left hemisphere laterality (i.e., LN and VSN). Moreover, as previously noted (Figley et al., 2015)—and as demonstrated by the number of long-range tracts with high connection counts—Euclidean distance between nodes was not the primary determinant of connection count.

In an effort to minimize the number of spurious fiber tracts (i.e., connections with low reproducibility) included in the final probabilistic atlases, group probability maps were only generated for tracts with connection counts of at least 8/32 (i.e., tracts for which at least one streamline was identified in ≥$\frac{1}{4}$ of the participants). Therefore, since no connections were found to meet this threshold in the AN or PVN, no probabilistic atlases were generated for these networks.

The resulting overall group probability maps—i.e., a superposition of all the individual ROI-to-ROI connections—for the remaining six networks are shown in Figure 2 (to the left of the connection counts) and in Supplementary Videos 9–14, while the individual tracts within each network (AN = 0, BGN = 3, LN = 5, PN = 1, SMN = 14, PVN = 0, HVN = 1 and VSN = 6; total = 30) are displayed in Supplementary Videos 15–44. Each of these probabilistic maps reflects the tract trajectories (i.e., locations) as a weighted average across participants, so they can be thresholded according to the desired amount of between-participant overlap (e.g., thresholding an image at 0.5 will show only those regions where at least 16/32 of the participants’ streamlines spatially overlap, etc.). As a corollary, the group probability maps are more conservative than the raw connection counts, which represent the number of participants who had at least one continuous streamline between two regions (in any location), while the group probability maps represent the proportion of participants who have overlapping streamlines at a particular spatial location. For this reason, values as low as 0 are possible (and indeed common) in the group probability maps, despite the requirement for each of them to have had a connection count greater than or equal to 8/32. Also, it should be noted that intensity-thresholding the probabilistic atlases will cause discontinuities to appear along tracts if, for example, voxels in the middle of a tract fall below the threshold.

As with our previous atlases (Figley et al., 2015), the current group probability maps have been coregistered to both MRIStudio and SPM coordinate systems (in order to account for spatial offsets between the JHU_MNI_SS and SPM8 templates) and saved as NIFTI images with 1 mm isotropic resolution for each individual tract and each network as a whole. These images, as well as the supplementary videos showing their 3D trajectories, can be freely downloaded from Version 2.0 of The UManitoba-JHU Functionally-Defined Human White Matter Atlas^5^.

**Figure 3:**
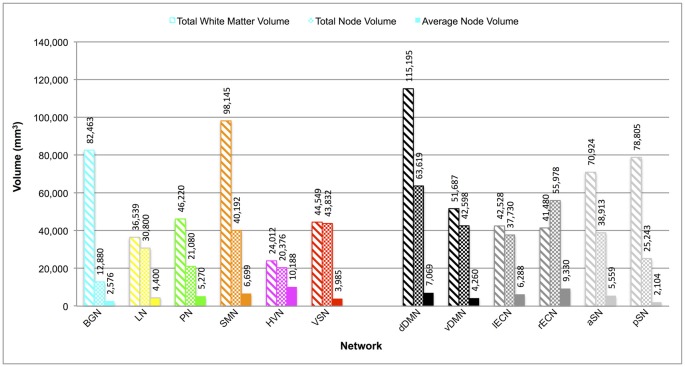
Total white matter volumes, total node volumes and average node volumes for each network. Total white matter volumes were determined by combining the functionally-defined group probability maps for all tracts with a connection count greater than or equal to 8/32 (but without any additional thresholding of the probability maps themselves). Note: total white matter volume did not appear to be correlated with total node volume (*r* = 0.29; *p* = 0.36) or average node volume (*r* = −0.25; *p* = 0.43) across networks.

The total white matter volumes of the overall group probability maps (unthresholded and in normalized MNI space), along with the total and average node (functional ROI) volumes of each network, are shown in Figure 3. Although the overall node volumes of the six new networks trended toward being smaller (*p* = 0.06; two-tailed *t*-test), both the average node volumes (*p* = 0.87) and the resulting functionally-defined white matter connectomes (*p* = 0.50) of the BGN, LN, PN, SMN, HVN and VSN were similar in size to those of the dDMN, vDMN, lECN, rECN, aSN and pSN (Figley et al., 2015). Interestingly, neither overall node volume (*r* = 0.29; *p* = 0.36) nor average node volume (*r* = −0.25; *p* = 0.43) were correlated with the volumes of the resulting white matter connectomes across networks.

**Figure 4:**
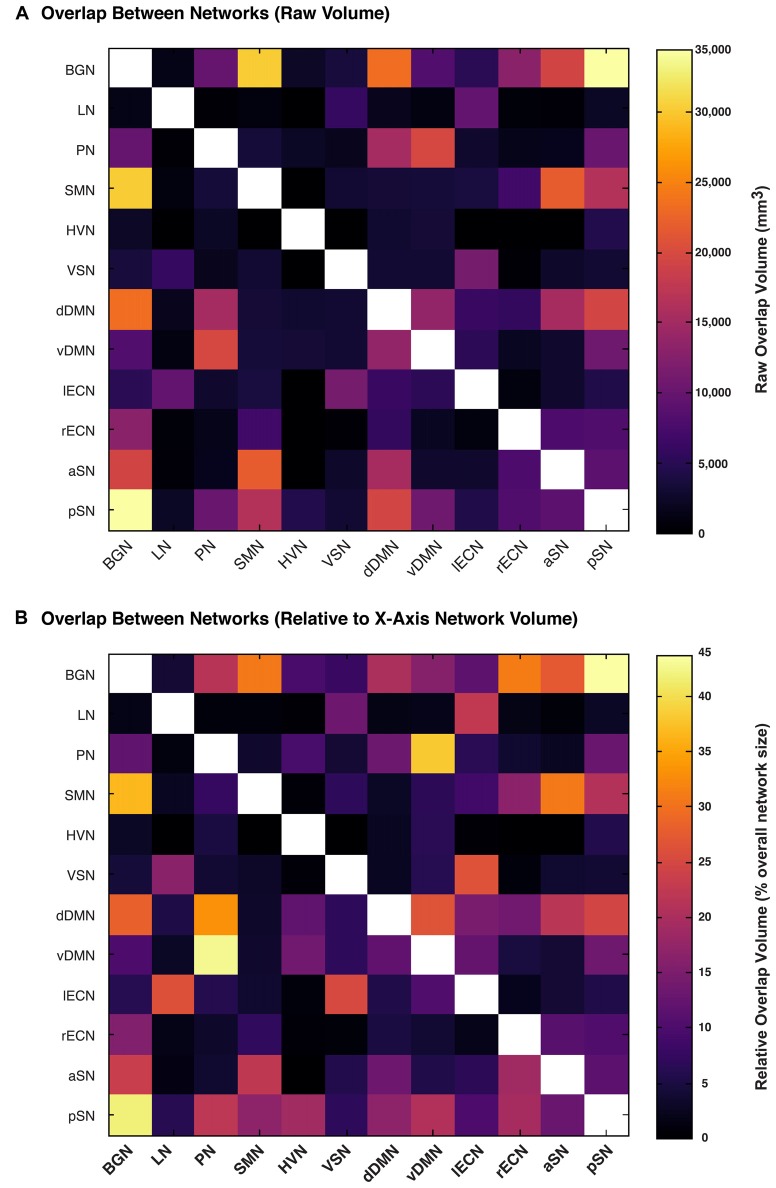
The amount of overlap between each functionally-defined white matter network (with the same masks used to calculate white matter volume in Figure 3). The amount of overlap between each pair of white matter networks is expressed **(A)** as a raw volume (in mm^3^), or **(B)** relative to the size of each network on the *x*-axis.

We then calculated the amount of spatial overlap between each of the six new white matter networks, as well as the six networks already reported in the first version of the UManitoba-JHU Functionally-Defined Human White Matter Atlas, with respect to each of the other eleven networks. The amount of overlap between each connectome was quantified both in terms of the absolute overlap volumes (Figure 4A) in mm^3^, and in terms of the relative overlap volume (Figure 4B) proportional to each network’s total white matter volume. Based on this, we observed that the largest overlaps in terms of absolute volumes (≥20,000 mm^3^) were observed for BGN vs. pSN (34,500 mm^3^), BGN vs. SMN (30,000 mm^3^), BGN vs. dDMN (23,500 mm^3^), SMN vs. aSN (22,000 mm^3^) and PN vs. vDMN (20,000 mm^3^). Based on the relative volumes (i.e., the proportion of the first network that overlaps with the second network), the largest overlaps (≥25% of the first network) were observed for pSN vs. BGN (44%), PN vs. vDMN (43%), BGN vs. pSN (42%), vDMN vs. PN (38%), BGN vs. SMN (37%), PN vs. dDMN (33%), SMN vs. BGN (31%), rECN vs. BGN (31%), aSN vs. SMN (31%), BGN vs. dDMN (28%), vDMN vs. dDMN (27%), aSN vs. BGN (27%), LN vs. lECN (26%), lECN vs. VSN (26%), VSN vs. lECN (25%) and pSN vs. dDMN (25%).

## Discussion

### General Discussion

Although several anatomically-defined white matter atlases, such as the JHU “Adam” and “Eve” atlases (Oishi et al., 2008, 2009), are already freely available, our group has taken a different approach by creating functionally-defined white matter atlases for various resting-state brain networks. Our previous article (Figley et al., 2015) systematically mapped white matter regions underlying the dorsal and ventral Default Mode Networks (dDMN and vDMN), left and right Executive Control Networks (lECN and rECN) and anterior and posterior Salience Networks (aSN and pSN). The current study now expands on that effort by including comprehensive white matter maps of the Basal Ganglia Network (BGN), Language Network (LN), Precuneus Network (PN), Sensorimotor Network (SMN), Higher Visual Network (HVN) and Visuospatial Network (VSN).

Based on our current understanding of the brain and how it is organized into distributed functional networks, we anticipate that these atlases will prove to be useful tools—in concert with quantitative white matter imaging methods like diffusion, magnetization transfer, and/or myelin water imaging—for future studies examining how structural connectivity differences between individuals or groups relate to task performance, clinical outcomes, etc. Our previous article (Figley et al., 2015) demonstrated how these functionally-defined white matter atlases can be used for voxel-wise and/or ROI-based analyses to examine relationships between structural measures throughout functionally-defined tracts or networks, and the initial white matter atlases have already proven useful for examining network-specific structural differences related to body composition (Figley et al., 2016) and Multiple Sclerosis (Ma et al., 2017). However, it is our hope that the addition of these new networks—related to different domains (e.g., language, vision, etc.)—will enhance the overall utility of the UManitoba-JHU Functionally-Defined Human White Matter Atlas.

### Differences between Anatomically-Defined and Functionally-Defined White Matter Tracts

Although the functionally-defined tracts identified in the current study will correspond to varying degrees with anatomically-defined white matter structures, an exhaustive comparison between anatomically- and functionally-defined tracts goes beyond the scope of the current manuscript. However, in order to illustrate how these functionally-defined atlases differ, we compared the language network LN1_LN4 connection (4068 mm^3^) from the current study to the left arcuate fasciculus (13,997 mm^3^), which was previously identified using the same data and analysis methods, but with anatomically-defined cortical ROIs (Figley et al., 2015). In addition to occupying only 29% of the total white matter volume of the anatomically-defined tract, we found that only 52% of voxels in the LN1_LN4 atlas overlapped with the arcuate fasciculus. Therefore, while approximately half of the voxels in the LN1_LN4 connection appear to be a subset of the nominal arcuate fasciculus, the other half are anatomically distinct.

### Why Were Primary Visual and Auditory Connectomes Not Identified?

Given that the PVN and AN represent two of the primary physical senses, and might therefore be assumed to have robust white matter connections, it seems surprising at first glance that no reproducible connections were identified. However, in the case of the PVN at least, the answer is actually quite simple. It turned out that although the resting state functional connectivity atlas for the PVN (Shirer et al., 2012)^6^ supposedly contained two nodes, one of the masks was actually an empty set (i.e., contained no voxels with a value >0), rendering it impossible for any diffusion streamlines to run between the two ROIs.

Unfortunately, there is not such a straightforward explanation for the lack of reproducible connections identified within the AN. Indeed, another recent study was able to identify white matter connections between bilateral Heschl’s gyri (Andoh et al., 2015) using slightly more sophisticated diffusion imaging parameters and probabilistic tractography. Therefore, the most likely explanation has to do with methodological differences, and each method has certain advantages and disadvantages regarding sensitivity vs. specificity tradeoffs (please see Study Limitations below for a more detailed discussion).

### Laterality vs. Bilateral Symmetry in Different Network Connectomes

Finding that the BGN and SMN show high degrees of bilateral symmetry (Figure 2) is perhaps not surprising given that the cortical nodes for both networks were bilaterally distributed and both networks have prominent roles in motor function and coordination—which include balanced control of both hemispheres/sides of the body. Our finding of a strongly left-lateralized language white matter connectome is also not surprising given that language is traditionally thought of as a left-lateralized function (Knecht et al., 2000), along with the notion that left-hemisphere regions tend to interact predominantly within the same hemisphere (Gotts et al., 2013). On the other hand, the predominantly left-lateralized VSN connectome is somewhat surprising, given that visuospatial processing has previously been thought to be mostly right-lateralized (Thiebaut de Schotten et al., 2011), although this can perhaps be partially explained by the theory that right-hemisphere regions and functions tend to be less connected within hemisphere due to strong interactions between hemispheres (Gotts et al., 2013).

### Overlap between White Matter Networks

One of the main observations from the network overlap analysis (Figure 4) was that there was substantial overlap between certain pairs of connectomes. Nonetheless, although certain white matter regions have been ascribed to multiple networks, many of these are consistent with their expected topologies. For example, since the Precuneus is one of the central nodes within the DMN, it is perhaps not surprising that the PN overlapped substantially (33% and 43%) with the dDMN and vDMN, respectively. Given that both the LN and lECN were highly left lateralized, it is also not surprising that a large proportion (26%) of the LN overlapped with the lECN. Finally, the BGN connectome—which overlapped with 44% of the pSN, 31% of the rECN, 31% of the SMN, 27% of the aSN, 22% of the PN and 20% of the dDMN—turned out to be the third largest by volume (Figure 3), and occupied substantial portions of central white matter structures. Therefore, it is not surprising that the BGN consistently overlapped with other networks, since any long-range projections through the same central white matter regions in those networks would quite likely lead to overlap (especially when examined at relatively poor spatial resolutions, such as those that are achievable with diffusion-based MRI approaches). Moreover, the BGN may have overlapping functional roles with many of the other networks, as the basal ganglia are known to be involved in motor function (SMN), executive function (lECN and rECN) and emotional regulation (aSN and pSN; Lanciego et al., 2012).

### Need for High-Dimensional Non-Linear Normalization and Accurate Coregistration

A point that was highlighted in our previous article (Figley et al., 2015), and one that bears repeating here, is that any future applications of these atlases for ROI or along-tract analyses will require either: (1) participant data (i.e., individual quantitative images such as FA maps, etc.) to be accurately warped and coregistered to the same template as our atlases (i.e., the SPM or MRIStudio MNI templates, which are distributed with our atlases for convenience); or (2) our functionally-defined white matter atlases to be accurately warped and coregistered to each individual participant’s native space. Importantly, previous analyses have shown that linear normalization is not sufficient to accurately align subcortical regions, including central white matter structures (Figley et al., 2015); therefore, in order for images to be “accurately warped”, high-dimensional, non-linear spatial normalization—e.g., using Advanced Normalization Tools (ANTs)^7^, Diffeomorphic Anatomical Registration Through Exponentiated Lie Algebra (DARTEL)^8^, fMRIB’s Nonlinear Image Registration Tool (FNIRT)^9^, Large Deformation Diffeomorphic Metric Mapping (LDDMM)^10^, etc. (see Klein et al., 2009)—is absolutely necessary.

### Study Limitations

A very detailed discussion of the pros, cons and limitations of the general methodology employed here was published in our original article (Figley et al., 2015), which is freely available (and in fact part of the same Frontiers Research Topic). Therefore, because the current manuscript used the same dataset and methods, we will not replicate that here in full. Instead, we will briefly highlight some of the main study limitations, and extrapolate on a few points based on new literature.

#### Limitations in Scope

One of the major limitations of our previous study and the existing functionally-defined white matter atlas is that we initially only included six functionally-connected brain networks (out of many such networks). These networks were chosen as a starting point because the dDMN, vDMN, lECN, rECN, aSN and pSN are among the most well-established and most-studied resting state brain networks. However, by creating similar atlases for the remaining networks in the Stanford resting state fMRI atlas (Shirer et al., 2012)^11^, we feel that the current study goes a long way to addressing this limitation.

Nonetheless, although fMRI studies have started to examine between-network connectivities (and it might very well be of interest to supplement these analyses with corresponding structural analyses), we have not yet generated any between-network white matter connectivity maps. It might be particularly interesting, for example, to create atlases for all of the connections between the dDMN and vDMN, lECN and rECN, aSN and pSN, and then between the combined DMN, ECN and SNs. However, because this is a combinatorial problem, where the total number of ROI-to-ROI connections (and therefore analysis time) increases drastically with the number of nodes-of-interest (either within or between networks), challenges like this become prohibitively time-consuming and labor-intensive using our current methods. Nonetheless, it should be noted that there are automated tractography tools, such as Freesurfer’s TRACULA (Yendiki et al., 2011) or AFNI’s FATCAT (Taylor and Saad, 2013), that could make such challenges more feasible. Alternatively, between-network investigations could be made more feasible by mapping connections between sub-sets of nodes that are likely to be involved in between-network interactions.

#### Limitations of a Modest Sample Size

Although the sample size of our dataset was consistent with previous DTI-based white matter atlases (Hua et al., 2008; Oishi et al., 2009), there are much larger diffusion MRI datasets, such as the Human Connectome Project (HCP)^12^, that are now freely available. Of course, any white matter connectome will depend on the individual(s) it was obtained from; and although we presume that 32 participants (16 male; 16 female) will yield relatively stable and generalizable white matter atlases, a larger sample size would allow a finer degree of thresholding of the final probabilistic atlases, and could enhance the apparent signal-to-noise in terms of true vs. spurious regions that are included in each network. Moreover, our atlases were created from a sample of healthy, middle-aged adults, and using a larger sample, such as the HCP data, would allow age-specific atlases to be generated (e.g., for pediatric and geriatric populations).

#### Limitations of Diffusion MRI

Diffusion-based MRI is non-invasive, provides 3D whole-brain coverage, and is therefore the only currently-available *in vivo* approach to estimate fiber trajectories between distributed human brain regions. Nonetheless, this approach has several limitations compared to histological staining and tract-tracing methods. In particular, diffusion imaging: (1) has orders of magnitude worse spatial resolution (Scherrer et al., 2012); (2) relies on an indirect measure of tissue microstructure (Mori and Zhang, 2006); (3) cannot reliably differentiate between myelinated, unmyelinated, or demyelinated fibers (Beaulieu, 2002); (4) cannot differentiate the directionality of fiber projections (i.e., afferent vs. efferent; Thomas et al., 2014); and (5) cannot, in many cases, even discriminate between monosynaptic and polysynaptic connections (Johansen-Berg and Rushworth, 2009).

Although the aforementioned limitations apply to all current diffusion MRI approaches—including high angular resolution diffusion imaging (HARDI; Tuch et al., 2002), Q-ball imaging (Tuch, 2004), and diffusion spectrum imaging (DSI; Wedeen et al., 2005)—we also note some additional limitations of DTI in particular, since this is the approach that was used to generate our atlases. For example, conventional tensor-based methods are not able to resolve complex fiber geometries (e.g., crossing, kissing, or turning fibers) nearly as well as more advanced fiber tracking techniques (Daducci et al., 2014). We therefore acknowledge that our deterministic, DTI-based connectomes are inherently biased toward Type-II (false-negative) errors, and that certain fibers and regions within the networks are more likely to have been excluded—as opposed to HARDI- and DSI-based methods, which tend to be biased toward higher Type-I (false-positive) errors, where aberrant fibers are sometimes included (Rodrigues et al., 2013).

Direct comparisons between deterministic diffusion tractography and gold-standard tract-tracing methods in rhesus macaques have revealed that diffusion-based connectome reconstructions generally produce reasonable estimates of large white matter projections (Dauguet et al., 2007; van den Heuvel et al., 2015). However, because tensor-based deterministic tractography approaches yield sparse connectomes (high specificity with comparatively low sensitivity) and higher-order probabilistic tractography approaches yield dense connectomes (high sensitivity with comparatively low specificity), other groups have begun to study tradeoffs between connectome sensitivity and specificity (Zalesky et al., 2016). Initial studies in this regard suggest (both empirically and theoretically) that “specificity is at least twice as important as sensitivity when estimating key properties of brain networks, including topological measures of network clustering, network efficiency and network modularity” (Zalesky et al., 2016). Therefore, although not perfect, the deterministic, tensor-based tractography approach used to generate our functionally-defined white matter atlases likely errs in the proper direction when it comes to connectome sensitivity vs. specificity tradeoffs.

Nonetheless, the fact remains that several real white matter connections were probably not identified by our tensor-based tractography analyses, so the current atlases cannot be used to draw conclusions about which regions are not part of a given tract or network. Rather, their intended use is to predict (with at least some measure of confidence) which white matter regions are part of a given tract or network, so that quantitative values can be extracted and compared between individuals or patient populations.

## Conclusion

Functional connectivity analyses within large-scale brain networks have become immensely popular, and are now ubiquitous throughout the cognitive neuroscience and neuroimaging literature. Yet, despite the fact that cerebral white matter forms a critical element that is necessary for these networks to “function” properly, comparable methods for assessing structural connectivity within these same networks have lagged far behind—in large part because the underlying white matter scaffolds have not been previously identified. To address this gap, we have used DTI and tractography to create functionally-defined white matter atlases (in stereotaxic coordinates) of the Basal Ganglia Network (BGN), Language Network (LN), Precuneus Network (PN), Sensorimotor Network (SMN), Higher Visual Network (HVN) and Visuospatial Network (VSN). It is our hope that this work will enhance the overall utility of our previously existing functionally-defined white matter atlases of the Default Mode, Executive Control and Salience Networks, and that it will provide a framework for future studies to evaluate white matter connectivity within these networks and attribute localized microstructural changes (either between individuals or groups) to particular functional brain networks, thereby providing deeper insights into the structural correlates of neural processes and/or diseases.

## Author Contributions

TDF, SMC and CRF conceived and designed the study, CRF and others (listed in the acknowledgments) acquired the data and all authors (i.e., TDF, BMM, NB, JK, SMC and CRF) were involved in the analysis and/or interpretation of the data. Furthermore, all authors (i.e., TDF, BMM, NB, JK, SMC and CRF) were involved in writing and revising the manuscript, and approved the final version for publication.

## Conflict of Interest Statement

The authors declare that the research was conducted in the absence of any commercial or financial relationships that could be construed as a potential conflict of interest.

## Footnotes

^1^http://www.nitrc.org/projects/uofm_jhu_atlas

^2^http://findlab.stanford.edu/functional_ROIs.html

^3^http://findlab.stanford.edu/functional_ROIs.html

^4^http://www.slicer.org

^5^http://www.nitrc.org/projects/uofm_jhu_atlas

^6^http://findlab.stanford.edu/functional_ROIs.html

^7^http://picsl.upenn.edu/software/ants/

^8^http://www.fil.ion.ucl.ac.uk/spm/

^9^https://fsl.fmrib.ox.ac.uk/fsl/fslwiki/FNIRT

^10^http://www.cis.jhu.edu/software/lddmm-volume/

^11^http://findlab.stanford.edu/functional_ROIs.html

^12^http://humanconnectome.org

## References

[B1] Andoh J. Matsushita R. Zatorre R. J. (2015). Asymmetric interhemispheric transfer in the auditory network: evidence from TMS, resting-state fMRI, and diffusion imaging. J. Neurosci. 35, 14602–14611. 10.1523/JNEUROSCI.2333-15.2015 26511249 PMC6605461

[B2] Beaulieu C. (2002). The basis of anisotropic water diffusion in the nervous system—a technical review. NMR Biomed. 15, 435–455. 10.1002/nbm.782 12489094

[B3] Beg M. F. Miller M. I. Trouve A. Younes L. (2005). Computing large deformation metric mappings via geodesic flows of diffeomorphisms. Int. J. Comput. Vis. 61, 139–157. 10.1023/b:visi.0000043755.93987.aa

[B4] Bressler S. L. Menon V. (2010). Large-scale brain networks in cognition: emerging methods and principles. Trends Cogn. Sci. 14, 277–290. 10.1016/j.tics.2010.04.004 20493761

[B5] Ceritoglu C. Oishi K. Li X. Chou M.-C. Younes L. Albert M.. (2009). Multi-contrast large deformation diffeomorphic metric mapping for diffusion tensor imaging. Neuroimage 47, 618–627. 10.1016/j.neuroimage.2009.04.057 19398016 PMC2857762

[B6] Daducci A. Canales-Rodríguez E. J. Descoteaux M. Garyfallidis E. Gur Y. Lin Y.-C.. (2014). Quantitative comparison of reconstruction methods for intra-voxel fiber recovery from diffusion MRI. IEEE Trans. Med. Imaging 33, 384–399. 10.1109/TMI.2013.2285500 24132007

[B7] Dauguet J. Peled S. Berezovskii V. Delzescaux T. Warfield S. K. Born R.. (2007). Comparison of fiber tracts derived from *in-vivo* DTI tractography with 3D histological neural tract tracer reconstruction on a macaque brain. Neuroimage 37, 530–538. 10.1016/j.neuroimage.2007.04.067 17604650

[B9] Djamanakova A. Tang X. Li X. Faria A. V. Ceritoglu C. Oishi K.. (2014). Tools for multiple granularity analysis of brain MRI data for individualized image analysis. Neuroimage 101, 168–176. 10.1016/j.neuroimage.2014.06.046 24981408 PMC4165692

[B10] Fedorov A. Beichel R. Kalpathy-Cramer J. Finet J. Fillion-Robin J.-C. Pujol S.. (2012). 3D Slicer as an image computing platform for the Quantitative Imaging Network. Magn. Reson. Imaging 30, 1323–1341. 10.1016/j.mri.2012.05.001 22770690 PMC3466397

[B11] Figley C. R. Asem J. S. A. Levenbaum E. L. Courtney S. M. (2016). Effects of body mass index and body fat percent on default mode, executive control, and salience network structure and function. Front. Neurosci. 10:234. 10.3389/fnins.2016.00234 27378831 PMC4906227

[B12] Figley T. D. Bhullar N. Courtney S. M. Figley C. R. (2015). Probabilistic atlases of default mode, executive control and salience network white matter tracts: an fMRI-guided diffusion tensor imaging and tractography study. Front. Hum. Neurosci. 9:585. 10.3389/fnhum.2015.00585 26578930 PMC4630538

[B13] Filley C. M. (1998). The behavioral neurology of cerebral white matter. Neurology 50, 1535–1540. 10.1212/wnl.50.6.1535 9633691

[B14] Gotts S. J. Jo H. J. Wallace G. L. Saad Z. S. Cox R. W. Martin A. (2013). Two distinct forms of functional lateralization in the human brain. Proc. Natl. Acad. Sci. U S A 110, E3435–E3444. 10.1073/pnas.1302581110 23959883 PMC3767540

[B15] Hua K. Zhang J. Wakana S. Jiang H. Li X. Reich D. S.. (2008). Tract probability maps in stereotaxic spaces: analyses of white matter anatomy and tract-specific quantification. Neuroimage 39, 336–347. 10.1016/j.neuroimage.2007.07.053 17931890 PMC2724595

[B16] Jiang H. van Zijl P. C. M. Kim J. Pearlson G. D. Mori S. (2006). DtiStudio: resource program for diffusion tensor computation and fiber bundle tracking. Comput. Methods Programs Biomed. 81, 106–116. 10.1016/j.cmpb.2005.08.004 16413083

[B17] Johansen-Berg H. Rushworth M. F. S. (2009). Using diffusion imaging to study human connectional anatomy. Annu. Rev. Neurosci. 32, 75–94. 10.1146/annurev.neuro.051508.135735 19400718

[B18] Klein A. Andersson J. Ardekani B. A. Ashburner J. Avants B. Chiang M.-C.. (2009). Evaluation of 14 nonlinear deformation algorithms applied to human brain MRI registration. Neuroimage 46, 786–802. 10.1016/j.neuroimage.2008.12.037 19195496 PMC2747506

[B19] Knecht S. Dräger B. Deppe M. Bobe L. Lohmann H. Flöel A.. (2000). Handedness and hemispheric language dominance in healthy humans. Brain 123, 2512–2518. 10.1093/brain/123.12.2512 11099452

[B20] Lanciego J. L. Luquin N. Obeso J. A. (2012). Functional neuroanatomy of the basal ganglia. Cold Spring Harb. Perspect. Med. 2:a009621. 10.1101/cshperspect.a009621 23071379 PMC3543080

[B21] Landman B. A. Farrell J. A. D. Jones C. K. Smith S. A. Prince J. L. Mori S. (2007). Effects of diffusion weighting schemes on the reproducibility of DTI-derived fractional anisotropy, mean diffusivity, and principal eigenvector measurements at 1.5T. Neuroimage 36, 1123–1138. 10.1016/j.neuroimage.2007.02.056 17532649 PMC12008999

[B22] Ma A. Y. Vitorino R. C. Hojjat S.-P. Mulholland A. D. Zhang L. Lee L.. (2017). The relationship between white matter fiber damage and gray matter perfusion in large-scale functionally defined networks in multiple sclerosis. Mult. Scler. [Epub ahead of print]. 10.1177/1352458517691149 28178867

[B23] Mazziotta J. C. Toga A. W. Evans A. Fox P. Lancaster J. (1995). A probabilistic atlas of the human brain: theory and rationale for its development. Neuroimage 2, 89–101. 10.1006/nimg.1995.1012 9343592

[B24] Mori S. Crain B. J. Chacko V. P. van Zijl P. C. M. (1999). Three-dimensional tracking of axonal projections in the brain by magnetic resonance imaging. Ann. Neurol. 45, 265–269. 10.1002/1531-8249(199902)45:2<265::AID-ANA21>3.0.CO;2-3 9989633

[B25] Mori S. Oishi K. Jiang H. Jiang L. Li X. Akhter K.. (2008). Stereotaxic white matter atlas based on diffusion tensor imaging in an ICBM template. Neuroimage 40, 570–582. 10.1016/j.neuroimage.2007.12.035 18255316 PMC2478641

[B26] Mori S. Zhang J. (2006). Principles of diffusion tensor imaging and its applications to basic neuroscience research. Neuron 51, 527–539. 10.1016/j.neuron.2006.08.012 16950152

[B27] Oishi K. Faria A. Jiang H. Li X. Akhter K. Zhang J.. (2009). Atlas-based whole brain white matter analysis using large deformation diffeomorphic metric mapping: application to normal elderly and Alzheimer’s disease participants. Neuroimage 46, 486–499. 10.1016/j.neuroimage.2009.01.002 19385016 PMC2885858

[B28] Oishi K. Zilles K. Amunts K. Faria A. Jiang H. Li X.. (2008). Human brain white matter atlas: identification and assignment of common anatomical structures in superficial white matter. Neuroimage 43, 447–457. 10.1016/j.neuroimage.2008.07.009 18692144 PMC2586008

[B29] Rodrigues P. Prats-galino A. Gallardo-Pujol D. Villoslada P. Falcon C. Prčkovska V. (2013). “Evaluating structural connectomics in relation to different Q-space sampling techniques,” in Proceedings Medical Image Computing and Computer-Assisted Intervention—MICCAI 2013, 16th International Conference, Part 1, eds Mori K. Sakuma I. Sato Y. Barillot C. Navab N. (Berlin, Heidelberg: Springer), 671–678. 10.1007/978-3-642-40811-3_84 24505725

[B30] Rosazza C. Minati L. (2011). Resting-state brain networks: literature review and clinical applications. Neurol. Sci. 32, 773–785. 10.1007/s10072-011-0636-y 21667095

[B31] Scherrer B. Gholipour A. Warfield S. K. (2012). Super-resolution reconstruction to increase the spatial resolution of diffusion weighted images from orthogonal anisotropic acquisitions. Med. Image Anal. 16, 1465–1476. 10.1016/j.media.2012.05.003 22770597 PMC3448812

[B32] Schmahmann J. D. Smith E. E. Eichler F. S. Filley C. M. (2008). Cerebral white matter: neuroanatomy, clinical neurology, and neurobehavioral correlates. Ann. N Y Acad. Sci. 1142, 266–309. 10.1196/annals.1444.017 18990132 PMC3753195

[B33] Shirer W. R. Ryali S. Rykhlevskaia E. Menon V. Greicius M. D. (2012). Decoding subject-driven cognitive states with whole-brain connectivity patterns. Cereb. Cortex 22, 158–165. 10.1093/cercor/bhr099 21616982 PMC3236795

[B34] Smith S. M. Vidaurre D. Beckmann C. F. Glasser M. F. Jenkinson M. Miller K. L.. (2013). Functional connectomics from resting-state fMRI. Trends Cogn. Sci. 17, 666–682. 10.1016/j.tics.2013.09.016 24238796 PMC4004765

[B35] Taylor P. A. Saad Z. S. (2013). FATCAT: (an efficient) functional and tractographic connectivity analysis toolbox. Brain Connect. 3, 523–535. 10.1089/brain.2013.0154 23980912 PMC3796333

[B8] Thiebaut de Schotten M. Dell’Acqua F. Forkel S. J. Simmons A. Vergani F. Murphy D. G. M.. (2011). A lateralized brain network for visuospatial attention. Nat. Neurosci. 14, 1245–1246. 10.1038/nn.2905 21926985

[B36] Thomas C. Ye F. Q. Irfanoglu M. O. Modi P. Saleem K. S. Leopold D. A.. (2014). Anatomical accuracy of brain connections derived from diffusion MRI tractography is inherently limited. Proc. Natl. Acad. Sci. U S A 111, 16574–16579. 10.1073/pnas.1405672111 25368179 PMC4246325

[B37] Tuch D. S. (2004). Q-ball imaging. Magn. Reson. Med. 52, 1358–1372. 10.1002/mrm.20279 15562495

[B38] Tuch D. S. Reese T. G. Wiegell M. R. Makris N. Belliveau J. W. Wedeen V. J. (2002). High angular resolution diffusion imaging reveals intravoxel white matter fiber heterogeneity. Magn. Reson. Med. 48, 577–582. 10.1002/mrm.10268 12353272

[B39] van den Heuvel M. P. de Reus M. A. Feldman Barrett L. Scholtens L. H. Coopmans F. M. T. Schmidt R.. (2015). Comparison of diffusion tractography and tract-tracing measures of connectivity strength in rhesus macaque connectome. Hum. Brain Mapp. 36, 3064–3075. 10.1002/hbm.22828 26058702 PMC6869766

[B40] van den Heuvel M. P. Hulshoff Pol H. E. (2010). Exploring the brain network: a review on resting-state fMRI functional connectivity. Eur. Neuropsychopharmacol. 20, 519–534. 10.1016/j.euroneuro.2010.03.008 20471808

[B41] Wedeen V. J. Hagmann P. Tseng W.-Y. I. Reese T. G. Weisskoff R. M. (2005). Mapping complex tissue architecture with diffusion spectrum magnetic resonance imaging. Magn. Reson. Med. 54, 1377–1386. 10.1002/mrm.20642 16247738

[B42] Xue R. van Zijl P. C. M. Crain B. J. Solaiyappan M. Mori S. (1999). *In vivo* three-dimensional reconstruction of rat brain axonal projections by diffusion tensor imaging. Magn. Reson. Med. 42, 1123–1127. 10.1002/(SICI)1522-2594(199912)42:6<1123::AID-MRM17>3.0.CO;2-H 10571934

[B43] Yendiki A. Panneck P. Srinivasan P. Stevens A. Zöllei L. Augustinack J.. (2011). Automated probabilistic reconstruction of white-matter pathways in health and disease using an atlas of the underlying anatomy. Front. Neuroinform. 5:23. 10.3389/fninf.2011.00023 22016733 PMC3193073

[B44] Zalesky A. Fornito A. Cocchi L. Gollo L. L. van den Heuvel M. P. Breakspear M. (2016). Connectome sensitivity or specificity: which is more important? Microb. Cell Fact. 142, 407–420. 10.1016/j.neuroimage.2016.06.035 27364472

